# Effect of probiotic and *Moringa oleifera* extract on performance, carcass yield, and mortality of Peking duck

**DOI:** 10.14202/vetworld.2022.694-700

**Published:** 2022-03-24

**Authors:** Widya Paramita Lokapirnasari, Bodhi Agustono, Mohammad Anam Al Arif, Lilik Maslachah, Evania Haris Chandra, Andreas Berny Yulianto

**Affiliations:** 1 Division of Animal Husbandry, Faculty of Veterinary Medicine, Universitas Airlangga, Indonesia; 2 Division of Veterinary Basic Medicine, Faculty of Veterinary Medicine, Universitas Airlangga, Indonesia; 3 Master Study program of Veterinary Agribusiness, Faculty of Veterinary Medicine, Universitas Airlangga, Indonesia; 4 Department of Veterinary Basic Medicine, Faculty of Veterinary Medicine, Wijaya Kusuma Surabaya University, Indonesia

**Keywords:** health, probiotic, *Moringa oleifera*, Peking duck

## Abstract

**Background and Aim:**

Antibiotics have been used as growth promoters in poultry. However, continuous and long-term antibiotics can cause resistance, suppress the immune system, and accumulate toxic residue. To overcome these problems, feed additives that are safe for livestock and health for humans are needed, including probiotics. Therefore, the study aimed to determine the effect of probiotics (*Lactobacillus acidophilus*, *Lactobacillus casei*, *Lactobacillus lactis*, and *Bifidobacterium* spp.) and *Moringa oleifera* extract on performance (body weight gain, body weight, feed intake, feed efficiency, and feed conversion ratio [FCR]), carcass yield (carcass weight and percentage of carcass) and mortality of Peking duck.

**Materials and Methods:**

This study used 48 Peking ducks, divided into four treatments and six replications. Each replication consisted of two ducks. The treatments were as follows: T0=control, T1=4 mL containing 1.2×10^8^ CFU/mL of probiotic in drinking water, T2=4 mL containing *M*. *oleifera* extract in drinking water, and T3=2 mL containing 1.2×10^8^ CFU/mL of probiotic in drinking water+2 mL containing *M*. *oleifera* extract in drinking water. The probiotics consist of *L*. *acidophilus*, *L*. *casei*, *L*. *lactis*, and *Bifidobacterium* spp. The data were statistically analyzed through analysis of variance. For the follow-up test, a multiple range test was conducted.

**Results:**

There was no significant difference (p>0.05) between body weight, feed intake, and mortality treatments. By contrast, control and treatment showed a significant difference (p<0.05) on feed efficiency, FCR, body weight gain, carcass weight, and percentage of carcass weight. Results of body weight gain statistics showed no significant difference (p>0.05) between T0 and T1, but T0 and T1 showed a significant difference with T2 and T3. The results of the feed efficiency statistic showed no significant difference (p>0.05) between T0, T1, and T2, but there was a significant difference between T0, T1, and T3. Feed efficiency at T2 showed no significant difference with T3, T1, and T0. The results of the FCR statistic showed no significant difference (p>0.05) between T0, T1, and T2, but there was a significant difference between T0, T1, and T3. FCR at T2 showed no significant difference with T3, T1, and T0. The carcass weight statistic showed no significant difference (p>0.05) between T0, T1, and T3, but there was a significant difference between T0 and T2. T2 showed no significant difference with T1 and T3. The carcass percentage statistic showed no significant difference (p>0.05) between T0 and T1, but T0 and T1 showed a significant difference (p<0.05) with T2 and T3.

**Conclusion:**

Based on the study results, it can be concluded that the use of a combination of probiotics (*L*. *acidophilus*, *L*. *casei*, *L*. *lactis*, and *Bifidobacterium* spp.) and *M*. *oleifera* extract can increase the production performance of Peking ducks and is safe for ducks’ health.

## Introduction

Duck is one of the livestock commodities that have a high economic value. The production of duck meat in Indonesia is always increasing. Asian countries, such as China, Vietnam, Indonesia, Malaysia, and Bangladesh, contribute significantly to the world’s duck population. In 2019, the world’s duck population was 1177.4 million heads [1]. In Indonesia, in 2017, the duck meat production was 42,318.86 tons, and then, it increased to 44,679.75 tons (in 2018) and 46,563.38 tons (2019) [2]. Peking duck is one type of duck with high meat productivity because globally, it tends to have the same performance as broilers in terms of body weight gain, feeds conversion ratio, and feed efficiency [3].

For decades, antibiotics have been used as growth promoters. However, continuous and long-term use of antibiotics can cause resistant pathogen strain development, suppression of the immune system, and accumulation of toxic residue. Thus, they have been banned in many countries [4,5], including Indonesia (Minister of Agriculture, Regulation of the Minister of Agriculture Number 14 [2017] concerning the Classification of Veterinary Drugs, Directorate of Animal Health, Directorate General of Livestock, and Animal Health Ministry of Agriculture of Indonesia, Indonesia). To overcome these problems, feed additives that are safe for livestock are needed, including probiotics. Probiotics are non-pathogenic living microorganisms that benefit the host through the intestinal microflora balance. Some probiotics that are often used to improve performance in poultry are *Bifidobacterium*, *Lactobacillus casei*, *Lactobacillus acidophilus*, and *Pediococcus pentosaceus* ABY 118 [6-9]. The content of appropriate and effective probiotic bacteria before consumption is recommended (10^8^-10^9^ CFU/g) so that an effective dose can be achieved in the large intestine (10^6^-10^7^ CFU/g) [10,11]. The functions of probiotics include increasing the immune system, protecting the gastrointestinal tract from pathogenic agents [12,13], lowering serum cholesterol levels [14], and increasing nutrient absorption [15,16]. In general, probiotics that are safe to use (generally recognized as safe) are *Lactobacilli*, *Bifidobacteria*, and *Saccharomyces* [17]. Several probiotics have been used to improve growth performance. They can be used as an alternative to growth promoter antibiotics, including the probiotics *Bifidobacterium* spp., *L*. *acidophilus*, *L*. *casei* WB 315, and *P*. *acidilactici* [6,7,18-20].

*Moringa oleifera* is one of such medicinal plants and contains many bioactive compounds [21,22]. The metabolite products or bioactive compounds in *M*. *oleifera* leaf extracts include flavonoids, steroids, tannins, saponins, phlobatannins, and terpenoids [23]. *M. oleifera* also contains proteins, vitamins, and minerals. Dried leaves contain high carotene (23.31-39.6 mg/100 g) of dry weight [24-26]. The predominant minerals in all tissues are iron, potassium, calcium, and magnesium [27]. *M*. *oleifera* leaf flour contained crude protein (28.7%), fat (7.1%), ashes (10.9%), carbohydrate (44.4%), calcium (3.0 mg/100 g), and iron (103.1 mg/100 g). In *M*. *oleifera* leaves, the tannin concentration is 20.7 mg/g [28], and kaempferol and quercetin range from 0.16-3.92 to 0.46-16.64 mg/g dry weight, respectively. The fatty acid in the leaves contains palmitic (16:0) and linolenic (18:3) acids [27]. Supplementation of fermented *M*. *oleifera* leaf powder on laying ducks can increase feed consumption, egg weight, and feed conversion ratio (FCR) [29].

This study aimed to determine the effect of probiotics (*L*. *acidophilus*, *L*. *casei*, *Lactococcus lactis*, and *Bifidobacterium* spp.) and *M. oleifer*a extract on the production performance (body weight gain, body weight, feed intake, feed efficiency, and FCR), carcass yield (carcass weight and percentage of carcass), and mortality of Peking duck.

## Materials and Methods

### Ethical approval

Ethical clearance of the study was approved by Animal Care and Use Committee Universitas Brawijaya (No.029-KEP-UB-2021).

### Study period and location

This study was conducted for 42 days (June-July 2021). The day-old ducks (DODs) were reared in the breeding farm located at a duck farm in Tulungagung Regency. Proximate analysis of the feed and variables examination were conducted at Laboratory of Animal Nutrition, Division of Animal Husbandry, Department of Veterinary Medicine Science, Faculty of Veterinary Medicine, Universitas Airlangga.

### Experimental design

The study material consisted of probiotics containing *L*. *acidophilus*, *L*. *casei*, *L*. *lactis*, and *Bifidobacterium* spp. (source of probiotics from W.P. Lokapirnasari’s and A.B. Yulianto’s collection) and 2 mL of *M*. *oleifera* extract in drinking water. *M. oleifera* extract was obtained through a modified method by Adedapo *et al*. [30]. The maceration process with 1:20 Aquadest for 12 h was used to obtain *M*. *oleifera*, which was then filtered to obtain the macerate and evaporated at a temperature of 40°C. The commercial feed contains crude protein (21%) and crude lipid (7%). This study used a complete randomized design, using 48 day-old duck (DOD) divided into four treatments and six replications, with each replication consisting of two DOD. The selection of dose is based on trial treatment. The treatments in this study were as follows: T0=control, T1=4 mL containing 1.2×10^8^ CFU/mL of probiotic in drinking water, T2=4 mL containing *M*. *oleifera* extract in drinking water, and T3=2 mL containing 1.2×10^8^ CFU/mL of probiotic in drinking water+2 mL containing *M*. *oleifera* extract in drinking water. The observed variables include body weight gain, feed intake, feed efficiency, FCR, body weight, carcass weight, percentage of carcass weight, and mortality. All variables were calculated with the following [31-33]. The experiment was conducted in 42 days. The rearing system was open with 12 h photoperiod regime, AI and coryza vaccinated, and *ad libitum* feeding management.

### Feed intake

The feed offered was weighed and recorded, as was the remaining feed, to determine the amount of feed intake. Feed intake was calculated by reducing the feed offered with the remaining feed, with the following equation:

Feed intake (g) = Feed offered (g) – remaining feed (g).

### FCR

The FCR was calculated by dividing the amount of feed intake by the body weight gain in that week, with the following equation:

FCR = Feed intake/body weight gain.

### Feed efficiency

The feed efficiency was calculated by dividing the amount of body weight gain by the feed intake in that week multiplied by 100, with the following equation:

Feed efficiency (%) = (body weight gain/feed intake) × 100.

### Body weight gain

Body weight gain was weighed at the beginning and end of the treatment phase. Body weight gain is the difference between initial weight and final weight during treatment, with the following equation:

Body weight gain (g) = final body weight (g) – initial body weight (g).

### Carcass weight

Carcass began with fasting for 12 h for emptying the feed in the digestive tract; then, slaughtering was performed by cutting the carotid artery, jugular vein, trachea, and esophagus; and duck’s feathers were removed by dipping in hot water for 35-45 s. According to Islamic law, the carcass is the body of poultry obtained after being slaughtered, including feather removal, viscera removal, and separation of the head, neck, and legs (shank).

### Carcass percentage

Carcass percentage was determined as the carcass weight with body weight and expressed as a percentage.

### Statistical analysis

The data were subjected to a homogeneity test inferential statistics (analysis of variance). The data were analyzed using Statistical Design for Social Sciences (SPSS) v.22 (IBM Corp., NY, USA). Duncan’s multiple range test was used for the follow-up test.

## Results

### Performance (body weight gain, body weight, feed intake, feed efficiency, and FCR)

The supplementation of the combination of probiotics and *M*. *oleifera* extracts showed no significant difference (p>0.05) between T0 and T1. Nevertheless, T0 and T1 showed a significant difference with T2 and T3 for body weight gain, but there was no significant difference (p>0.05) in body weight (Table-1). The highest body weight gain was found in probiotics and *M*. *oleifera* extract, which was not different from *M*. *oleifera* extract treatment. The lowest body weight gain was found in control, which was not different from giving probiotics only. The result of body weight showed the same for all treatments.

**Table-1:** Average body weight gain and body weight.

Treatment	Body weight gain (g/duck/day) and standard deviation	Body weight (g/duck/day) and standard deviation
T0	61.43^a^±1.21	3175.40^a^±78.72
T1	61.74^a^±1.49	3202.40^a^±136.03
T2	63.93^b^±1.86	3177.60^a^±78.25
T3	64.98^b^±1.63	3219.80^a^±86.61

^a,b^Means in the same column, with different superscripts, represent significant differences between treatments

The supplementation of the combination of probiotics and *M*. *oleifera* extract showed a significant difference (p<0.05) between treatments for feed efficiency. The results of the feed efficiency statistic showed no significant difference (p>0.05) between T0, T1, and T2, but there was a significant difference between T0, T1, and T3. Feed efficiency at T2 showed no significant difference with T3, T1, and T0. The supplementation of the combination of probiotics and *M*. *oleifera* extract was significantly different (p<0.05) between treatments for FCR. The results of the FCR statistic showed no significant difference (p>0.05) between T0, T1, and T2, but there was a significant difference between T0, T1, and T3. FCR at T2 showed no significant difference with T3, T1, and T0, but the results of feed intake showed no significant difference (p>0.05) in feed intake between treatments (Table-2). The results indicated the same feed intake for all treatments. The combination of probiotics and *M*. *oleifera* extract showed the highest feed efficiency value, whereas the lowest was found in control. The value of feed efficiency in the treatment of probiotics only and *M*. *oleifera* only gave the same good feed efficiency value. The best FCR value was in the combination of probiotics and *M*. *oleifera* extract, whereas the lowest was found in control. The FCR value in the probiotic treatment only and *M*. *oleifera* only gave the same good FCR value.

**Table-2:** Average of feed intake, feed efficiency, and feed conversion ratio.

Treatment	Feed intake (gram/duck/day) and standard deviation	Feed efficiency (%) and standard deviation	FCR
T0	200.59^a^±0.59	30.63^a^±0.65	3.27^b^±0.06
T1	200.06^a^±2.50	30.87^a^±1.02	3.24^b^±0.11
T2	202.79^a^±2.03	31.53^ab^±0.87	3.17^ab^±0.09
T3	202.36^a^±3.68	32.11^b^±0.764	3.12^a^±0.07

^a,b^Means in the same column, with different superscripts, represent significant differences between treatments

### Carcass yield (carcass weight and carcass percentage) and mortality

The combination of probiotics and *M*. *oleifera* extract supplement showed a significant difference (p<0.05) between carcass weight and carcass percentage treatments. The results of the carcass weight statistic showed no significant difference (p>0.05) between T0, T1, and T3, but there was a significant difference between T0 and T2. T2 showed no significant difference between T1 and T3. The results of the carcass percentage statistic showed no significant difference (p>0.05) between T0 and T1, but T0 and T1 showed a significant difference (p<0.05) with T2 and T3. The supplementation of the combination of probiotics and *M*. *oleifera* extract showed no significant difference (p>0.05) on mortality (Table-3). The highest carcass weight production was found in the combination of probiotics and *M*. *oleifera* extract, probiotic only, and *M*. *oleifera* extract only. By contrast, the lowest output of carcass weight was found in the control. The highest carcass percentage value was found in the combination treatment of probiotics and *M*. *oleifera* and the treatment of *M*. *oleifera* only. By contrast, the lowest carcass percentage was only in probiotic treatment and control. Mortality in all treatments showed the same good results.

**Table-3:** Average carcass weight, carcass percentage, and mortality.

Treatment	Carcass weight (gram/duck) and standard deviation	Carcass percentage (gram/duck) and standard deviation	Mortality (%)
T0	2123.10^a^±26.13	66.32^a^±0.72	0.00^a^±0.00
T1	2140.90^ab^±80.71	67.19^a^±1.08	0.00^a^±0.00
T2	2229.50^b^±62.65	70.16^b^±1.03	0.00^a^±0.00
T3	2199.30^ab^±77.40	68.91^b^±1.26	0.00^a^±0.00

^a,b^Means in the same column, with different superscripts, represent significant differences between treatments

## Discussion

This study aimed to determine the effect of probiotics (*L*. *acidophilus*, *L*. *casei*, *L*. *lactis*, and *Bifidobacterium* spp.) and *M*. *oleifera* extract on the production performance (body weight gain, body weight, feed intake, feed efficiency, and FCR), carcass yield (carcass weight and percentage of carcass), and mortality of Peking duck.

### Performance (body weight gain, body weight, feed intake, feed efficiency, and FCR)

The results indicated that the highest body weight gain was found in T3 and T2, which differed from T1 and T0 treatments (Table-1). This study revealed that the probiotic concentration of 1.2×10^9^ used has the appropriate viability. The viability of probiotics is needed to function appropriately in the digestive tract [34]. The results are in line with other studies that proved that the use of probiotics *Lactobacillus* and *M*. *oleifera* in broilers could positively affect growth performance [18]. The body weight gain of ducks is influenced by feed intake, and the increase in feed intake would undoubtedly be followed by body weight gain if there are no physiological disorders in the digestive tract of the ducks [35].

Furthermore, it indicated no significant difference in feed intake between treatments (Table-2). It is because lactic acid bacteria are feed additives whose mechanism of action is to balance the microbial composition of the digestive tract. Other influencing factors, among others, that is, the energy and protein content of the feed for each treatment, were the same. Consequently, the feed consumption of each treatment showed no significant difference between the treatments. Thus, improvement in body weight gain in treatment groups T3 and T2 might be due to the better feed efficiency and nutrient availability in *M*. *oleifera* as a source of essential nutrients for better performance.

The combination of probiotics and *M*. *oleifera* extract showed a higher feed efficiency value (T3 and T2) than the control (T0) (Table-2). Feed efficiency is related to the FCR. The results showed that supplementation of the combination of probiotics and *M*. *oleifera* extract (T3) could improve FCR values compared with controls (Table-2). FCR describes feed efficiency in livestock. The combination of probiotics and *M*. *oleifera* extract showed that the amount of feed consumed was the same but resulted in more significant body weight gain than the control.

The FCR is influenced by several factors, including digestibility, quality and nutritional content of feed, environment, and genetics. The FCR values for T3, T2, and T1 were 3.12, 3.17, and 3.24, respectively, indicating a higher improvement in FCR than in other studies. Another study on local duck supplemented with probiotic 10^8^ CFU showed an FCR value of 5.7 [36]. The other study showed that using probiotic Lactina containing L*. bulgaricu*s, L*. helveticu*s, L*. acidophilu*s, L*. lacti*s, *Enterococcus faeciu*m, and *Streptococcus thermophilus* in mule ducks can reduce the concentration of *Escherichia col*i (67.47%) and *Salmonell*a (54.54%) and also increase the concentration of *Lactobacillu*s (82.89%) in the cecum, increase body weight, decrease FCR by 4%, and decrease mortality [37].

Our study showed that using a combination of probiotics and M*. oleifer*a extract is in line with the results of the previous study [29] shows that supplementation of fermented M*. oleifer*a can improve FCR (3.27-3.52) in ducks compared with controls (3.81). Supplementation of probiotics and *M*. *oleifera* extract can improve feed efficiency and FCR. It is believed to be related to the content of alkaloids. Alkaloids are nitrogen-containing organic compounds in plants derived from amino acid metabolism [38,39] to help duck health produce optimal production. Probiotic supplementation in the ratio also affects the FCR value. The ducks given probiotic supplementation in the ration can utilize food substances properly because of the help of fiber-digesting bacteria in the digestive tract, which affects body weight. In addition, probiotics help establish a microflora balance in the digestive tract to maintain the host’s health [40]. The positive effect of forming a microflora balance in the digestive tract is feed consumption, and feed conversion becomes more efficient [41].

### Carcass yield (carcass weight and percentage) and mortality

The results revealed that the lowest carcass weight was in control, whereas T2, T3, and T1 produced a higher carcass weight than the control (Table-3). The combination of probiotics and *M*. *oleifera* extract resulted in carcass weight in T2, T3, and T1. A higher carcass weight also results in a higher carcass percentage. Furthermore, the results revealed that the T3 and T2 produced a higher carcass percentage (68.91-70.16%) than the control (66.32%). The rapid growth of livestock will affect the final weight so that it affects the percentage of carcass weight. The results indicated that the body weight obtained follows the market needs of Peking ducks in the range of 3.0-3.3 kg body weight on 38-40 days of age [33]. Supplementation of probiotics and *M*. *oleifera* extract was in line with other studies, which showed that the probiotics *Lactobacillus fermentum* and *E*. *faecium* had no significant effect (p>0.05) on body weight and feed intake in ducks [42].

Supplementation of probiotics and *M*. *oleifera* extract in this study showed a higher carcass percentage value compared with other studies (Table-3). The use of probiotics *L*. *fermentum*, *L*. *acidophilus*, and *Bacillus* spp. showed a carcass percentage value of 60.21-61.19% [43]. The use of probiotics in other studies showed a carcass percentage of 58.93% [44]. The higher carcass percentage was due to the combination of probiotics and *M*. *oleifera* extract, which could increase the digestibility of nutrients, affecting body weight and carcass weight. In addition, *M*. *oleifera* leaf contains phytochemical properties such as antioxidants and antimicrobial that can improve the health of broiler chickens.

The percentage increase of the carcass is related to the bacteriocin produced by probiotics to inhibit the growth of pathogenic bacteria. Probiotics also have enzymes to degrade proteins and carbohydrates into amino acids, N, and dissolved carbon, requiring protein synthesis. Increased protein digestibility affects the improvement of protein metabolism so that it affects the increase in meat protein synthesis [43]. The results align with Khattab’s study, which showed that adding probiotics *L*. *acidophilus* and *L*. *casei* in the diet showed an increase in body weight gain and final body weight and also an increase in intestinal enzyme activity (protease, lipase, and amylase), morphometric (goblet cell count, villi length, and crypt depth), and an increase in carcass percentage [45]. The mechanism of the action of probiotics is to stick or adhere to and colonize in the digestive tract, producing antimicrobial substances helping the enzymatic digestion of feed [46]. Supplementation of probiotics and *M*. *oleifera* extract in this study showed no mortality. It indicates that probiotics and *M*. *oleifera* extract are safe to use for livestock. Probiotics must meet the requirements of generally being recognized as safe [17]. These results are consistent with other studies, which showed that the use of probiotics *L*. *fermentum* and *E*. *faecium* did not cause mortality in ducks [43].

## Conclusion

Based on the revealed results, it was concluded that the use of a combination of probiotics 2-4 mL (containing 1.2×10^8^ CFU/mL) of probiotics (*L*. *acidophilus*, *L*. *casei*, *L*. *lactis*, and *Bifidobacterium* spp.) and 2 mL of *M*. *oleifera* in drinking water in broiler ducks could be used to improve production performance (body weight gain, feed efficiency, FCR, carcass weight, percentage of carcass weight, and zero mortality). Therefore, the future scope of this study is that probiotics and *M*. *oleifera* extract can be applied to improve production performance in broiler ducks.

## Authors’ Contributions

WPL and ABY: Contributions to conception and design of the study and drafted and revised the manuscript. MAAA and LM: Analysis and interpretation of the data. EHC: Collected the data. BA: Drafted and revised the manuscript. All authors read and approved the final manuscript.
